# A holistic approach to the age validation of *Mullus barbatus* L., 1758 in the Southern Adriatic Sea (Central Mediterranean)

**DOI:** 10.1038/s41598-018-30872-1

**Published:** 2018-09-05

**Authors:** Pierluigi Carbonara, Simona Intini, Jerina Kolitari, Aleksandar Joksimović, Nicoletta Milone, Giuseppe Lembo, Loredana Casciaro, Isabella Bitetto, Walter Zupa, Maria Teresa Spedicato, Letizia Sion

**Affiliations:** 1 COISPA Tecnologia & Ricerca, Stazione Sperimentale per lo Studio delle Risorse del Mare, via dei Trulli 18-20, 70126 Bari, Italy; 2 University of Bari Department of Biology Aldo Moro, LRU CoNISMa, via Orabona 4, 70125 Bari, Italy; 3 Agriculture University of Tirana Aquaculture & Fishery Laboratory, Lagje Nr. 4. Rr. Skënderbeg, 2001 Durres, Albania; 4 Institute of Marine Biology, Dobrota bb, P.O. box 69, Kotor, 85330 Montenegro; 5 FAO AdriaMed Project c/o FIAF (room C620), viale delle Terme di Caracalla, 00153 Rome, Italy

## Abstract

The growth of *Mullus barbatus* has been widely studied using different methods, but no previous study has focused on age validation. The uncertainty in estimating the age of the red mullet by otolith reading is linked to the number of false-growth increments laid down before the annulus. The capture of red mullets in the early life stage allowed us to estimate their size at the metamorphosis from the pelagic to the demersal phase. The comparison between the metamorphosis size and the back-calculated length of the first growth increment clarified the position of the false growth increment on the otolith. Moreover, the analyses of the otolith marginal increments in adult and juvenile specimens allowed us to define the deposition patterns of their annuli. The modal components of the length–frequency distribution analysis (LFDA) were identified in the winter survey (ELEFAN and Bhattacharya methods), and they did not show significant differences from the length back-calculation of the annuli. Moreover, no significant differences were found between the growth curves calculated by otolith reading (back-calculation and direct otolith reading) and the LFDA. The agreement between the length–frequency results and the otolith age estimation either corroborated or indirectly validated the growth pattern estimated in the otoliths of the red mullet, mainly when the direct validation methods (e.g. mark-recapture, captivity, radiochemical) were difficult to implement, like the case of this species. The comparison of the results of the present work to previous Mediterranean studies showed agreement with the slow growth pattern.

## Introduction

Red mullet (*Mullus barbatus* L., 1758) is a benthic species that inhabits the sandy and muddy bottoms of the continental shelf. The species has a widespread geographical distribution that extends from the eastern Atlantic along the European and African coasts to the Mediterranean Sea and the Black Sea. The habitat ranges from the shallow littoral coasts down to 300 m although depths between 20 and 200 m^1^ are preferred. This species is characterised by gregarious behaviour^2^.

Since red mullet has a great commercial value, it is a main target species of coastal fisheries in the Mediterranean. Accordingly, it is subject to regular stock assessment, which has provided evidence of its overfishing status in most geographical Mediterranean sub-areas^3–6^. This condition needs to be managed by measures that include the accurate evaluation of the productive potential of the stocks, which is closely connected to the growth profile of the species.

Although red mullet is one of the most studied species in the Mediterranean context, some aspects of its growth and age estimation are still controversial. According to the literature, *M.barbatus* is reported to have an average length at the first year between 7.54 cm^7^ and 18.93 cm^8^. This variation in length could not be exclusively explained by geographic variability and genetic differences. Moreover, other factors, such as age estimation methods and age estimation criteria, could have contributed to this high discrepancy.

Scales and otoliths are direct methods employed in the age estimation of red mullet. However, the otolith reading has been considered the most suitable method^9^ because scale reading may cause the underestimation of older ages of this species^9,10^. The interpretation of otolith growth zones of red mullet is challenged by many factors, such as the occurrence of false growth increments in addition to those formed annually, the deposition of the reproductive growth increment, and the overlapping of the annuli in older specimens^9,11,12^. One of the most important reported sources of discrepancies between readers is the identification of the first annulus^9,10^.

In studying this species, a main problem is that direct age validation methods (e.g. mark-recapture, captivity rearing and radiochemical dating)^13^ are quite difficult to be applied, because of the high mortality after capture (stress, scale loss and wounds)^14^ and the short life span of the species^15–17^. Uncertainty in age determination and in the estimate of growth parameters has a considerable effect on the results of stock assessments results because uncertainties about the first annulus can lead to the over- or under-estimation of one year in age determination is important in a species that has a life span of 5–8 years^15–17^.

In this study, samples were collected in Geographical Sub-Area (GSA) 18 (the South Adriatic Sea) during fishery-independent and fishery-dependent surveys (i.e. MEDITS trawl survey and biological sampling in the context of the Data Collection Framework [DCF] [EU Reg. 199/2008], respectively). The results of the marginal analysis, the marginal increment analysis, the morphological analysis, the back-calculation and the length–frequency distribution analysis (LFDA) (ELEFAN and Bhattacharya methods) were combined to develop a holistic approach to age estimation validation. Thus, based on the results of different methods, combined with observations of the early life-stage, controversial aspects of the otolith age estimation in the red mullet were addressed.

## Materials and Methods

### Sampling

**Figure 1:**
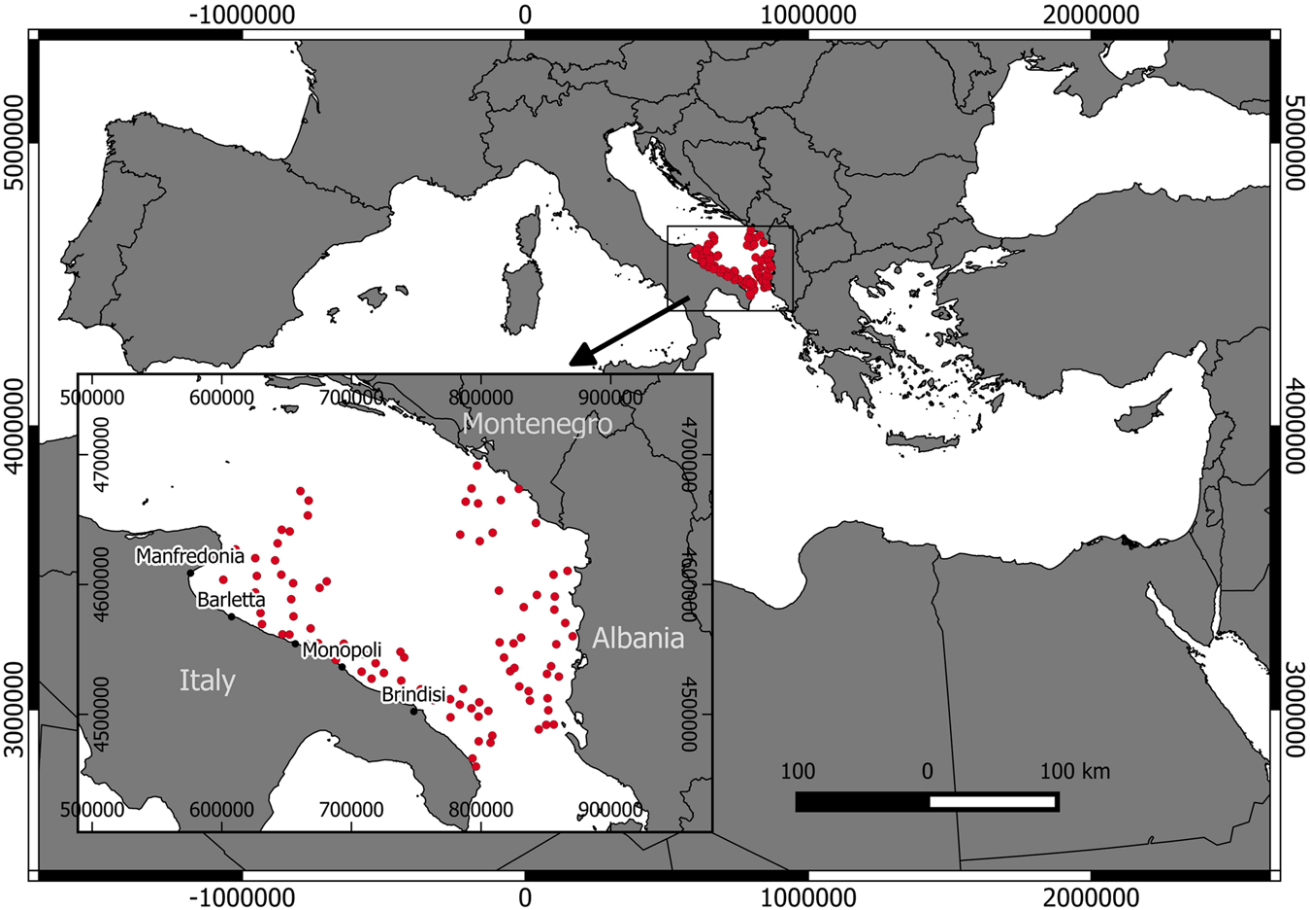
Geographical allocation of the hauls carried out in the MEDITS and GRUND trawl surveys in the GSA 18 (Southern Adriatic Sea). Main fishing ports along the south Adriatic coasts of Italy (in the window).

In the period from 2011–2016, red mullet samples were collected monthly at commercial landings through discard monitoring (DCF; EU Reg. 199/2008) in the fishing ports along the Italian Southern Adriatic coasts (GSA18; Fig. 1). Additional samples were obtained from the Mediterranean International Trawl Survey (MEDITS), which was conducted from 2009–2016^18^ in the South Adriatic Sea, including Albania and Montenegro, and from the national trawl survey GRUND^19^ (January 2009). The sampling protocol used in the MEDITS trawl survey was used also in the GRUND survey even to allocation the sampling stations (Fig. 1).

The following biological parameters were analysed: total length (TL) to the nearest 0.5 cm and sex. The unsexed juvenile specimens were divided into two sexes using the sex *ratio* value of the first fully sexed class (9 cm).

### Deposition pattern

The deposition pattern of the annuli on the otolith was analysed by a semi-direct method: Marginal Analysis (MA) was the qualitative approach; Marginal Increment Analysis (MIA) was the quantitative approach. The marginal analysis considered the monthly evolution of the type of edge (transparent or opaque) of the otolith. The two-edge types were defined when more of the ¾ of the margin appeared transparent or opaque. The otoliths in which about 50% of the edge was opaque or transparent were not considered in the analysis. The analysis was conducted in two separate groups: juveniles with a TL (<TL_25_) ranging from 3.5–8.0 cm and adults with a TL (>TL_75_) ranging from 13–22 cm^20^. The MIA considered the mean monthly marginal increment. The Relative Marginal Distance (RMD) was estimated in each otolith analysed following the equation reported in Panfili *et al*.^21^ as the ratio between the last mark from the edge, Absolute Marginal Distance (AMD) and the latter completed annulus and the distance separating the two last marks (Di, i − 1):

$${\rm{RMD}}={\rm{AMD}}/{{\rm{D}}}_{{\rm{j}}-1}$$

The MIA was restricted to only a few age groups (II and III age classes) to avoid the influence of seasonal differences among the age classes on the entire sample^13^.

### Morphological traits

**Figure 2:**
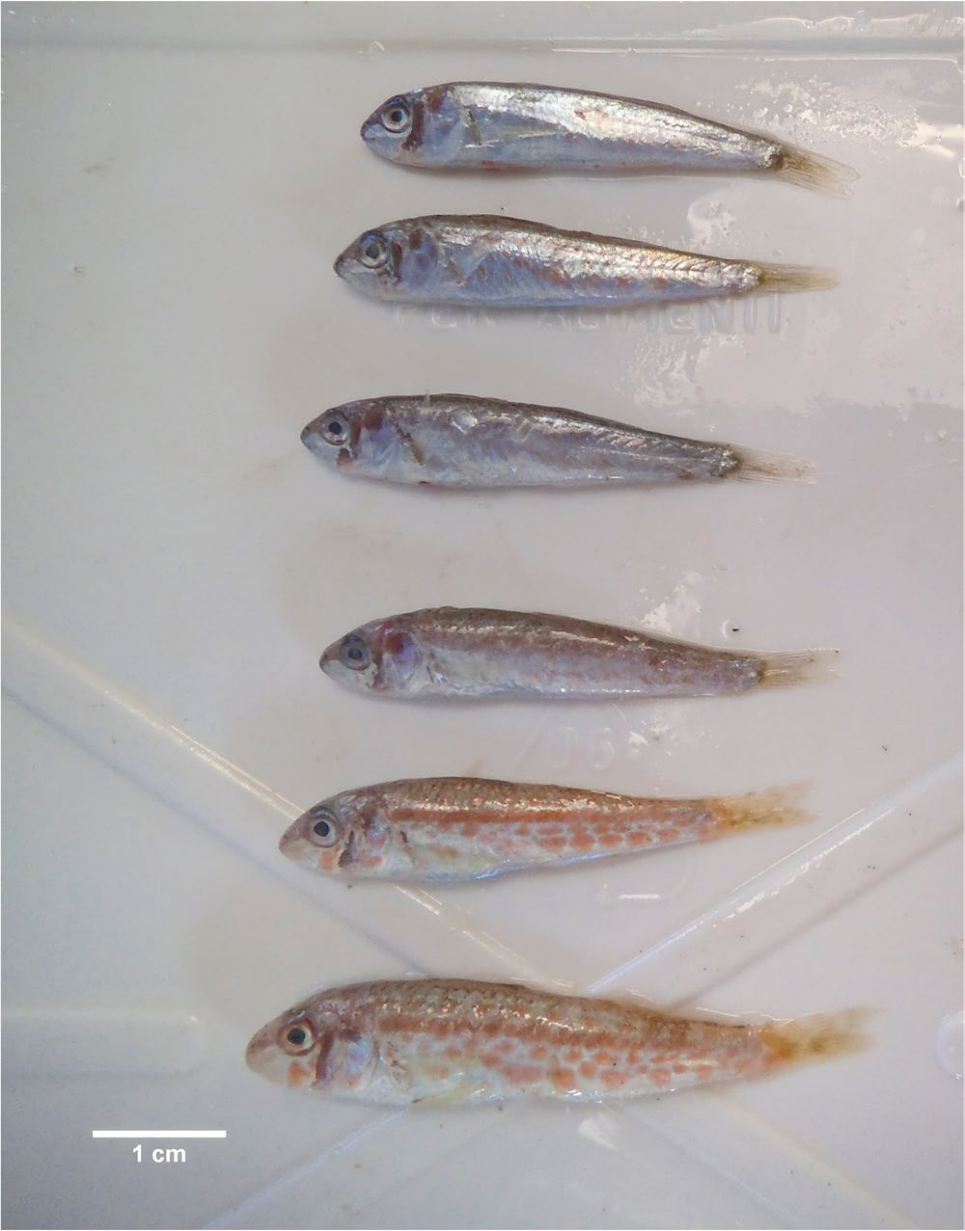
Blue (pelagic) and red (demersal) *Mullus barbatus* juveniles caught during the MEDITS trawl survey (2012).

Two types of juvenile red mullet (blue pelagic and red demersal) were caught in some hauls of the MEDITS surveys in 2011 and 2012 due to the high vertical opening of the MEDITS net^18^. (Fig. 2). The juveniles were classified as *M. barbatus* following the morphological trait reported in Vasil’eva^22^. For each specimens the TL to the nearest 1 mm were collected. The sizes of 50% of the juvenile specimens that had red demersal patterning was calculated using a binomial Generalized Linear Model (GLM^23^) with a logistic link.

### Otolith age estimation

**Figure 3:**
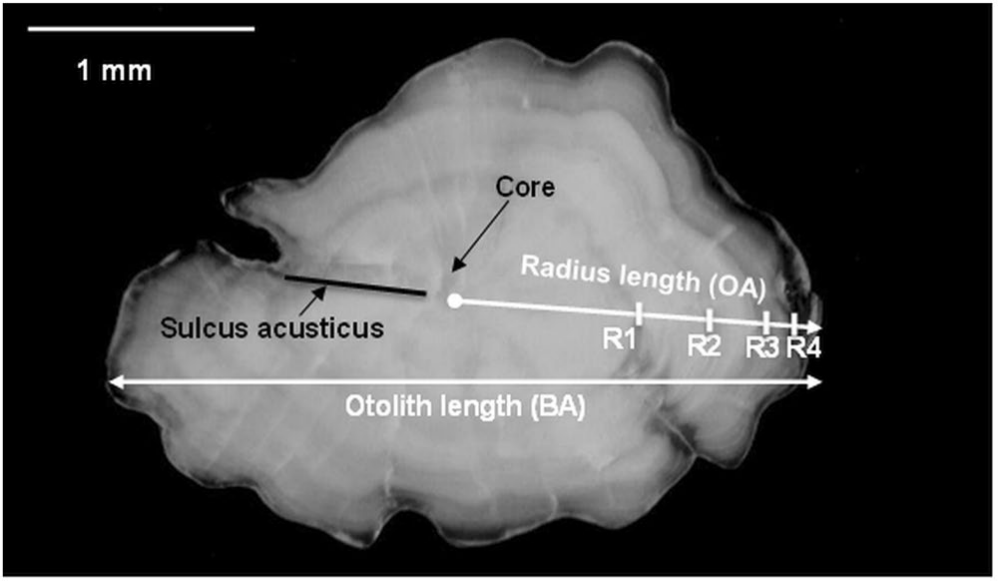
Definition of the measurements of the red mullet otolith.

Sagitta otoliths were collected from a subsample of specimens captured during the trawl MEDITS 2013–2016 (1,334 individuals) and from the commercial and discard samplings from 2011–2016 (5,769 individuals). Both otoliths (right and left) were removed in at least five specimens of both sexes and in each length class (0.5 cm) in the monthly time series. In total, 7,103 otoliths, preferably the right one, were read: 3,950 females and 3,153 males. In subsamples of otoliths, morphometric measurements and annuli distances were routinely taken. The nature of the edge (i.e. opaque or transparent) was always noted. Several morphometric data were collected from the nucleus (Fig. 3): otolith length (BA), otolith radius length (OA), type of edge (transparent or opaque) and annuli distance (R1, R2….Rn). All measurements were taken in the posterior area on the distal side along the longitudinal axis joining the sulcus and the nucleus^11^ (Fig. 3). The measurements were taken from the right otolith of only annuli that were clearly defined according to the criteria proposed by ICES^9^. A linear regression analysis^23^ was used to investigate the relationships between the TL vs. BA and TL vs. OA (Fig. 3). Moreover, the relationship between BA and TL in the juveniles (182 specimens, including TL between 35 to 73 mm) was analysed to calculate the otolith length at hatching (see the section on back-calculation).

The otoliths were rinsed with seawater and analysed using a binocular microscope with light reflected against a black background. In the analysis, the best otolith orientation was with the distal surface turned up and the proximal surface (sulcus acusticus) turned down (Fig. 3)^11^. Because the otoliths of *M. barbatus* are thin, they do not need to be rinsed before the age analysis and positioning them in sea water for a long time could make them transparent and therefore difficult to read. Instead, in the bigger specimens (TL > 20 cm) a short placement in seawater (2–4 minutes) was sufficient for their reading. The transparent zone (dark = slow growth) followed by the opaque zone (white = fast growth) is considered an annual increment (annulus). The age estimation was done assuming that the annulus is constituted by alternating the deposition of one transparent band with an opaque one. The age was assessed by counting the transparent growth increments^11^. In the age estimation, the criteria reported in ICES^9,11^ were used to recognize the annuli with a resolution of half year^24^.

One of the most important points of good practice in the age analysis is to adopt a standardized age estimation scheme^25^. According to reproductive patterns, the theoretical birthday was set at 1^st^ July^20^. The age estimation scheme utilized is reported in Table 1. The scheme takes into account the deposition pattern of the annuli based on the quarterly resolution^24^. This scheme considers several elements: the number of annuli, the theoretical birthday, the date of capture, the age resolution (half year) and the edge type (opaque or transparent)^24^. Considering that the monthly deposition pattern of the annulus is the first part of the year (1st and 2nd quarters), most specimens analysed presented the transparent edge. Moreover the transparent edge does not represent one year spent, but half year, considering July 1^st^ as date of birth. In this case, the age was equal to the number of the annuli included in the edge minus 0.5 years.

**Table 1:** The age estimation scheme for *M. barbatus* with the theoretical birthday 1^st^ July.

Date of capture	Otolith edge	Age
1st Quarter	Opaque	n + 0.5
	Transparent	N − 0.5
2nd Quarter	Opaque	n − 0.5
	Transparent	N − 0.5
3rd Quarter	Opaque	n
	Transparent	N
4th Quarter	Opaque	n
	Transparent	N − 1

**n** is the number of transparent growth increments excluded the edge; **N** is the number of transparent growth increments included the edge.

During the first part of the year, we also found specimens with an opaque edge. In the first quarter, it may be the case that specimens have not yet begun to lay down the transparent growth increment, whereas in the second quarter, specimens have already started to lay down the summer growth increment (opaque). Therefore, in the first quarter, the age was equal to the number of transparent growth increments (n) plus 0.5 years. In second quarter, the age was equal to the number of transparent growth increments (n) minus 0.5 years.

In the second part of the year (i.e. the 3rd and 4th quarters), most specimens presented an opaque edge in accordance with the monthly deposition pattern of the annulus. Consequently, because the set date of birth (1^st^ July) was passed, the age was equal to the number of transparent growth increments (n). In the rest of the specimens with transparent edges in the second part of the year in the 3rd quarter, we surmised that they had not yet begun to lay down the opaque growth increment. In the 4rd quarter, the specimens had already started to lay down the transparent winter-growth increment. Therefore, during the 3rd quarter, the age was equalled to the number of transparent growth increments, including the edge (N). In the 4rd quarter, the age was equalled to the number of growth increments included the edge N minus 1 year (N − 1).

### Growth

Growth was described according to the standard von Bertalanffy growth function^26^:

$${{\rm{TL}}}_{{\rm{t}}}={{\rm{TL}}}_{\infty }\ast [1-{{\rm{e}}}^{\mbox{--}{\rm{k}}\ast ({\rm{t}}\mbox{--}{\rm{t}}0)}]$$

where TL_t_ is the total length at age t, TL_∞_ is the predicted asymptotic length (infinity), k is the growth coefficient and t_0_ is the prenatal length. A non-linear least squares regression procedure was used to estimate the parameters of the von Bertalanffy growth function (VBGF) using length at age pairs and minimizing the sum of the squared residuals between observed and expected values (GLM^23^).

The growth performance index Φ′^27^ was calculated as follows:

$${\rm{\Phi }}^{\prime} =\,\mathrm{log}\,{\rm{k}}+2\,\mathrm{log}\,{\rm{TL}}\infty $$

This parameter represents a synthetic index of von Bertalanffy function calculated by the growth parameters L∞ and k; it widely used to compare the overall growth performance of different species and/or stocks of the same species.

### Back calculation method

The fish length at which different transparent annuli were deposited was back-calculated separately for the two sexes using the biological intercept procedure, which is known as the Campana formula^28^:

$${{\rm{TL}}}_{{\rm{i}}}={{\rm{TL}}}_{{\rm{c}}}+({{\rm{TL}}}_{{\rm{c}}}-{{\rm{TL}}}_{{\rm{o}}})\ast ({{\rm{OL}}}_{{\rm{i}}}\,\mbox{--}\,{{\rm{OL}}}_{{\rm{c}}})/({{\rm{OL}}}_{{\rm{c}}}\,\mbox{--}\,{{\rm{OL}}}_{{\rm{o}}})$$

where TL_i_ and OL_i_ are fish length and otolith length, respectively, at age i; TL_c_ and OL_c_ are fish length and otolith length, respectively, at capture; TL_0_ and OL_0_ are fish length and otolith length, respectively, at hatch (biological intercept). A biologically based intercept corresponds to the beginning of proportionality between fish and otolith growth. This point corresponds to the time of hatching^28^. In red mullet, the length at hatch is an average of 2 mm^29,30^. In this study, the value of otolith length at hatching was calculated using the linear relationship between OL and TL in the smaller juveniles (182 specimens), including TL between 35 and 73 mm. Thus, the effect on the TL-OL relationship^28^ of the growth rate of the older specimens was minimized. An age was assigned to each back-calculated TL following the above-mentioned criteria in order to calculate the parameters of the von Bertalanffy growth function (TL_∞_, k and t_0_) using the non-linear least squares regression procedure.

### Length–frequency distribution analysis

The LFDs were based on data collected in GRUND (2009) and MEDITS (2009–2016) surveys. The Bhattacharya method, which is incorporated in the FISAT software^31^, was used to discriminate the normal distribution assuming that each mode in the overall size-frequency distribution represented a cohort. The separation index among different cohorts was taken into account, and values < 2 indicated a large overlap between cohorts, which was considered unacceptable^31^.

The estimation of growth parameters was performed also using the Electronic Length Frequency Analysis I (ELEFAN I) routine, which is incorporated in the FISAT software^31^. ELEFAN I restructures the LFD in valleys and peaks by assigning positive values to length classes that contain many animals and small or negative values to length classes that contain few animals^32^. The fit scores (Rn) were calculated by summing the values of the length classes through which each growth curve passed. The growth curve accumulated a high fit score passing through length classes (or modes) with large numbers of animals. The growth curve with the highest score was considered the best estimate. ELEFAN I estimates only two of the three growth parameters (TL_∞_ and k); thus, the third parameter (t_0_) was calculated by Pauly’s equation^33^:

$$\mathrm{Log}(-{{\rm{t}}}_{0})=(0.3922)-0.2752\,\mathrm{log}\,{{\rm{TL}}}_{\infty }-1.038\,\mathrm{log}\,{\rm{k}}$$

The LFDs of GRUND 2009 were sliced to fit a finite mixture distribution model using the mixdist R Cran package^34^. The LFDs were expressed as the sum of normal distributions: one for each age class and separately for the two sexes. The ELEFAN growth parameters were used in the optimization algorithm. Hence, the mean total length and standard deviation by age class were derived.

### Statistical analysis

The monthly mean values of RDM were statistically tested using Tukey’s post hoc test of the ANOVA^23^. The linear relationships between the TL and the measurements of the otoliths were statistically tested using the analysis of variance of regression (ANOVA). Moreover, the relationships between the TL and BA by sex were compared (slope and intercept) through ANCOVA (p < 0.05) in order to assess the differences in growth between sexes. In the juvenile specimens, the ANOVA^23^ was used to statistically test the linear relationship between otolith length and TL. Moreover, the VBGF growth curves obtained in this study (otolith reading, LFDA) were statistically compared using the Chen-test^35^.

The mode components (cohort) of the GRUND LFD were obtained in the winter period when the deposition of the transparent growth increments occurred. For this reason, the mean length of the cohorts was identified using the Bhattacharya method and ageing slicing (ELEFAN I). The results were compared to the mean TL back-calculated from the transparent growth increments that were identified during the otolith analysis using the Kruskal-Wallis non-parametric test.

The means of the growth performance index Φ′, grouped by method (otolith, LFDA and scale). Mediterranean sub-regions (i.e., Western Mediterranean, Central Mediterranean and Eastern Mediterranean), were statistically compared using the Kruskal-Wallis non-parametric test and a relative post hoc test to determine the differences among the groups.

### Compliance with ethical standards

All specimens of red mullet (*Mullus barbatus*) analysed in this study were collected from the fishery (Data Collection Framework [DCF]; EU Reg. 199/2008). Therefore, this study does not comply with the European Commission recommendations (Directive 2010/63/EU of the European Parliament and of the Council of 22 September 2010) or with Italian National Law (Decree Law n. 26 of 4 March 2014) on the protection of animals used for scientific experiment. In the cases where the animal was alive when it arrived on the vessel during the scientific survey (MEDITS – DCF, EU Reg. 199/2008), it was suppressed by administering an overdose of anaesthetic in compliance with the recommendation of Decree Law n. 26 of 4 March 2014. All efforts were made to minimize suffering. The protocol used during all experiments was approved by the Ethics Committee of COISPA (Italian Ministry of Health 15/2015-UT).

## Results

**Figure 4:**
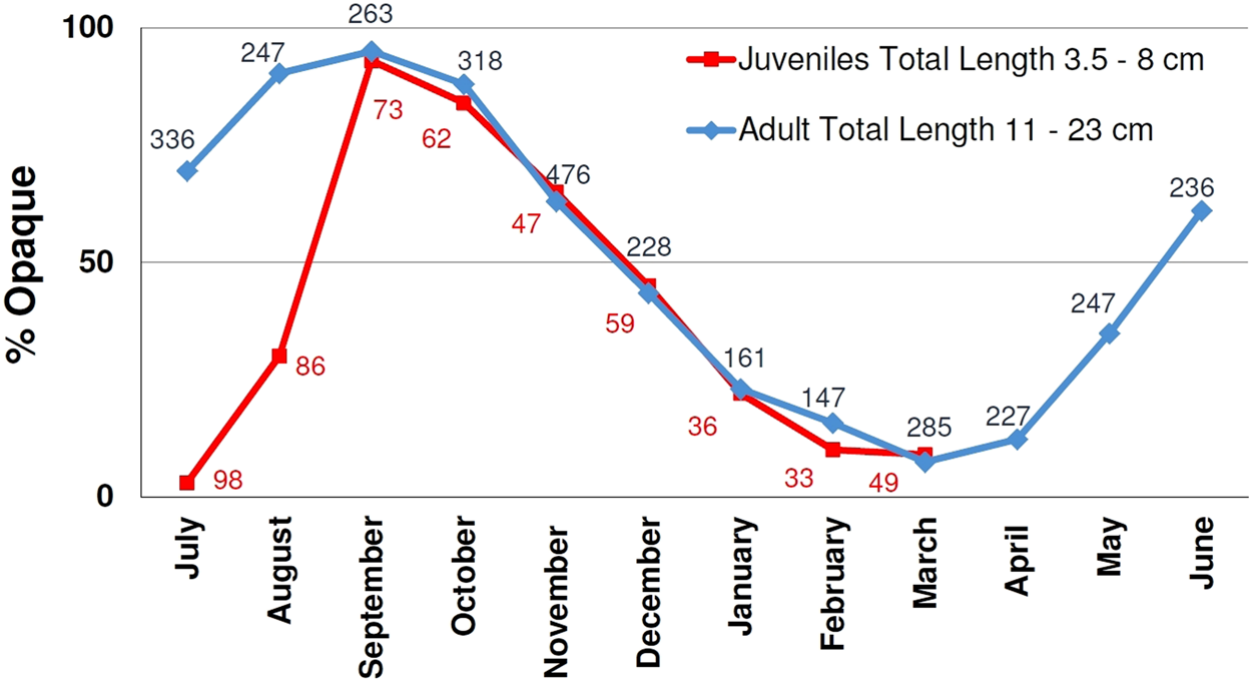
Monthly percentage (%) (MA) of opaque margins in red mullet *sagittae*. The blue trend represents the adult, while the red one the juveniles. Numbers of specimens used to calculate the percentage by month is also indicated.

The margin monthly evolution (MA) in the adult specimens showed a prevalence of the opaque edge (>50%) between June and November while the transparent edge is prevalent from December to May (Fig. 4). Following this pattern of annulus deposition, yearly one transparent area is followed by one opaque one. Whilst, in the juveniles specimens the deposition of a transparent growth increment during the summer months (July and August) was observed. For the rest of the year, the adults and juveniles specimens seemed to have the same deposition annulus patterns (Fig. 4).

These results demonstrated the deposition of only one false annulus before the first winter annulus in the juvenile specimens.

**Figure 5:**
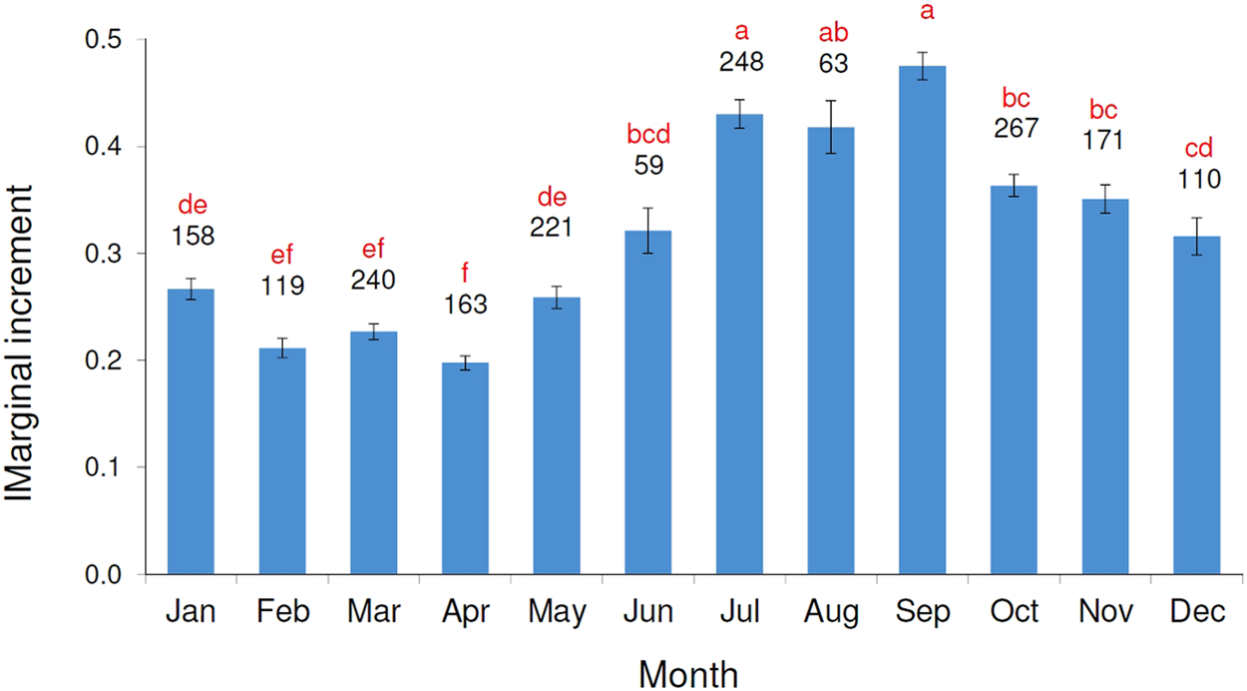
Mean monthly marginal increment (MIA) for red mullet otoliths. Numbers indicate sample size; the bars represent the standard error of the mean; the same letter show the absences of significant differences (Tukey’s post hoc test of ANOVA P < 0.05).

The MIA showed the same pattern of the MA with significantly higher marginal increments in the summer months (July–September) and significantly lower marginal increments in the winter and early spring (February–April) (Fig. 5). These results showed that the growth of otolith was significantly higher (i.e. quantitative approach) during the deposition of the opaque area (i.e. qualitative approach).

**Figure 6:**
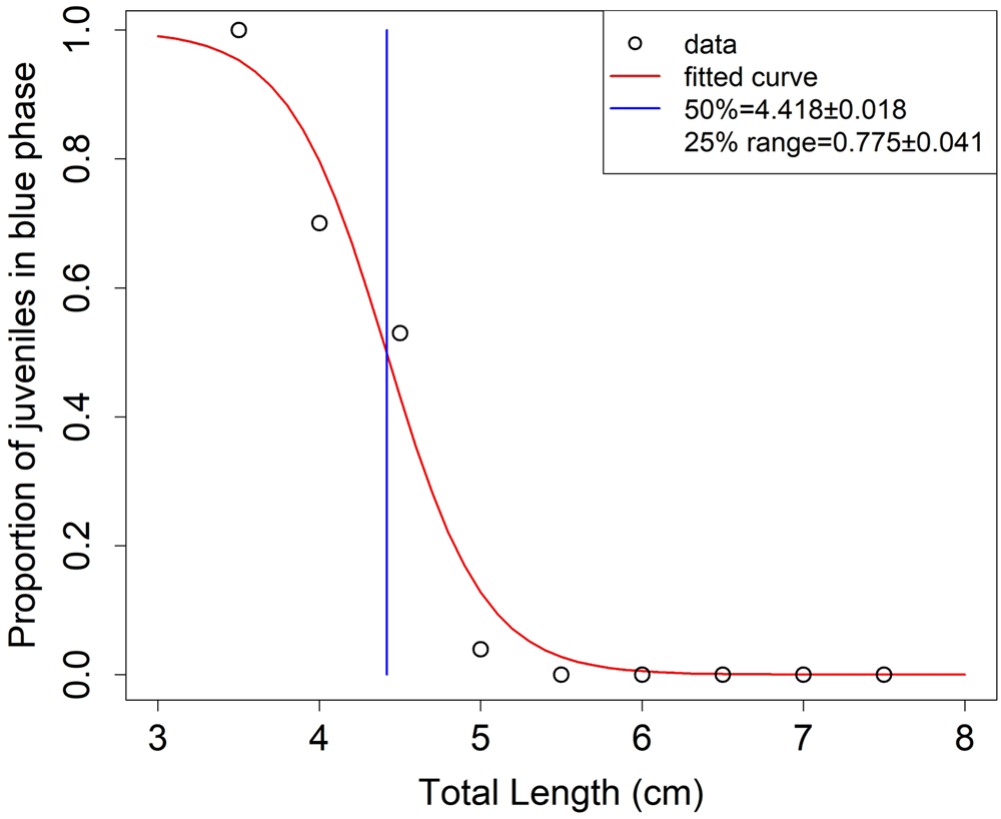
The logistic curve of the proportion of red demersal juveniles of red mullet by length. The length where the 50% of the specimens showed demersal coloration and 25% range (25–75%) was calculated.

In two hauls during the MEDITS survey in 2011 and 2012, two types of juveniles (blue pelagic and red demersal) were caught. The total of 2,202 specimens were caught with TL included between 3.5 and 7.5 cm. Figure 6 illustrates the percentages by length class (0.5 cm) of the two juvenile types. The results of the logistic model analysis indicated that the length where the 50% of the specimens showed demersal coloration was at 4.4 cm (Fig. 6). The smallest red specimen observed was 4 cm in TL, while the first length class with 100% of demersal specimens was 5.5 cm.

**Figure 7:**
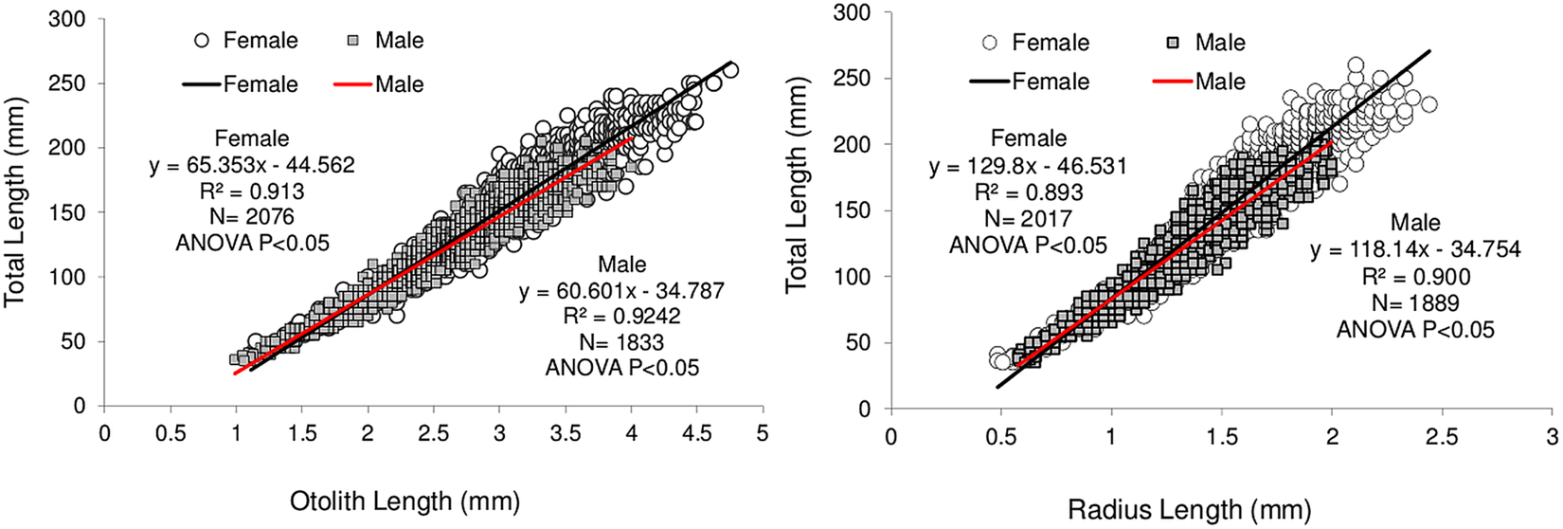
Linear regression between fish total length, otolith length (right) and otolith radius (left) for female and male (left) of red mullet. The equation, R^2^, number of specimens and statistical results are also reported.

The otolith morphometric descriptors (otolith length [OL] and otolith radius [OR]) and fish total length (TL) were significantly linearly correlated in both sexes (linear regression P < 0.05) (Fig. 7). Moreover, the comparison between sexes showed significant differences in a higher intercept and slope in the females (ANCOVA p < 0.05).

**Figure 8:**
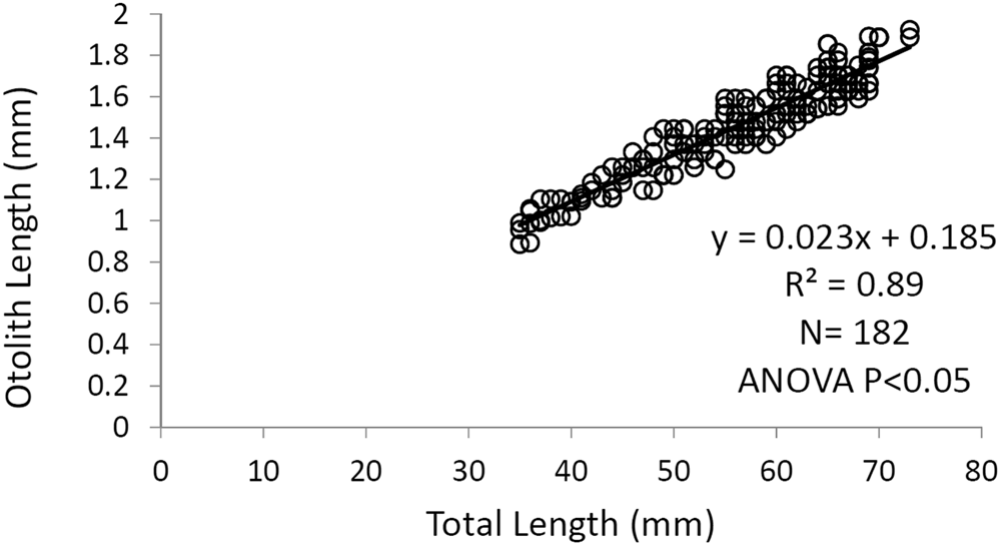
Linear regression between OL and TL (juveniles ranged between 35 and 73 mm) used to calculate the otolith at hatching. The equation, R^2^, number of specimens and statistical test are also reported.

The significant linear relationship between OL and TL (P < 0.05) (Fig. 8) of the juveniles (specimens with TL included between 35 to 73 mm) allowed the estimation of otolith length at hatching (0.23 mm) by using the mean TL at the hatching of red mullet 2 mm in length^29,30^.

**Figure 9:**
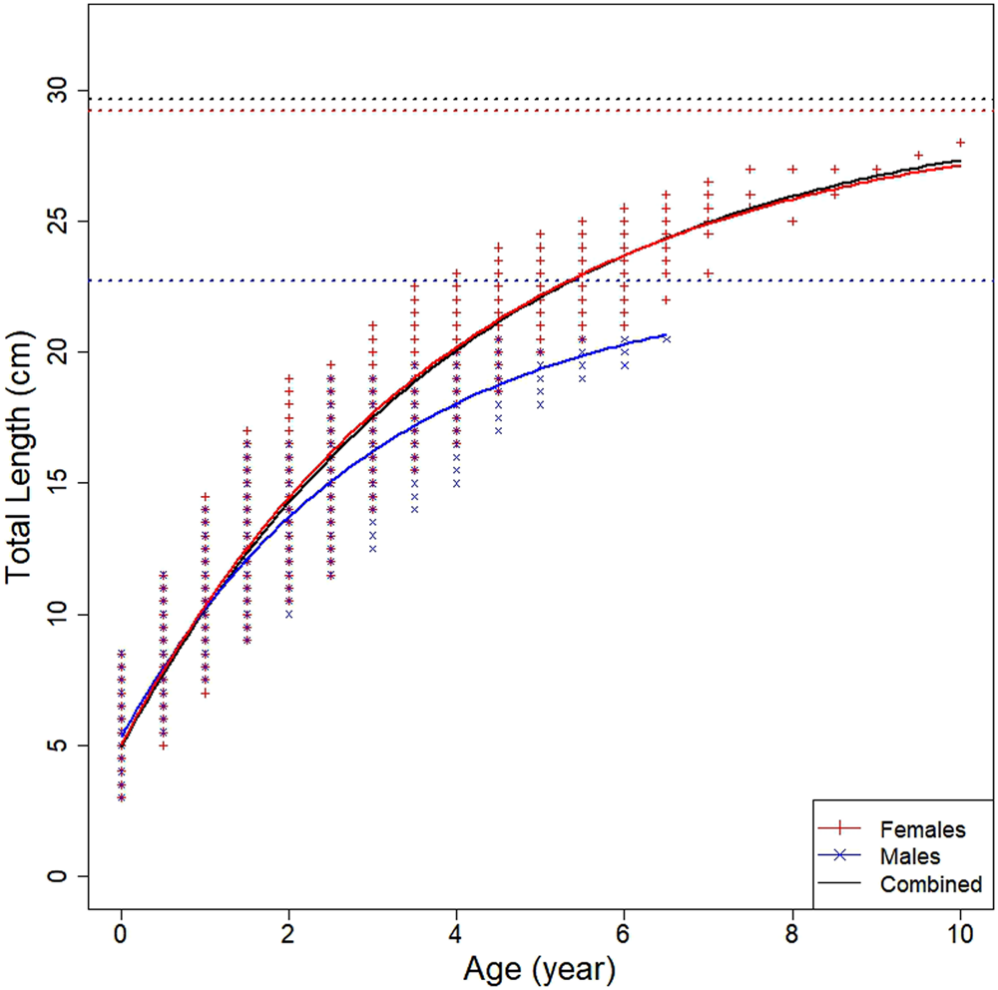
Growth curves obtained by the Von Bertalanffy growth equation for females (red), male (blue) and combined sex (black).

The fish were aged, and the age classes were from I to XI for the females and from I to VII for the males. The maximum observed total length was 28 cm TL in the females and 20.5 cm in the males TL in the age classes XI and VII. The growth parameters obtained by direct aging were the following: L_∞_ = 29.185 cm, k = 0.247 year^−1^ and t_0_ = −0.768 year for females; L_∞_ = 22.725 cm, k = 0.328 year^−1^ and t_0_ = −0.816 year for males; L_∞_ = 29.649 cm, k = 0.237 year^−1^ and t_0_ = −0.769 year for sex combined (Fig. 9).

Considering the linear correlation between body length (TL) and otolith length (Fig. 8), the length at hatching^29,30^ and the estimation of the otolith length at hatching through the Campana formula^28^ were back-calculated from the fish lengths (Tables 2 and 3) corresponding to the transparent growth increments recognised on the otolith.

**Table 2:** Mean back-calculated length for each growth increment for female red mullets collected in the Southern Adriatic Sea. SD = standard deviation; CV = coefficient of variation.

N° Growth Increments	N° Specimens	Growth Increments								
		1°	2°	3°	4°	5°	6°	7°	8°	9°
1	97	4.5								
2	556	5.1	9.6							
3	652	4.5	9.3	12.3						
4	462	5.1	9.8	13.0	15.4					
5	233	4.5	9.9	13.1	15.6	17.4				
6	83	4.4	10.1	13.3	15.9	17.8	19.4			
7	26	5.1	10.0	13.2	15.6	17.5	19.1	20.6		
8	5	4.5	9.8	12.3	14.8	17.6	18.5	19.9	21.0	
9	3	4.6	9.7	13.3	15.17	17.6	20.2	21.8	22.9	23.9
Tot. number	2117	2109	2020	1464	812	350	117	34	8	3
Mean (cm)		**4.51**	**9.32**	**12.73**	**15.49**	**17.49**	**19.29**	**20.58**	**21.69**	**23.90**
Mean increment (cm)		5.71	3.61	3.41	2.76	2.00	1.80	1.30	1.11	2.20
SD		0.76	1.01	1.17	1.20	1.25	1.18	1.25	1.41	0.60
CV		1.33	1.08	0.92	0.78	0.71	0.61	0.61	0.65	0.25

**Table 3:** Mean back-calculated length for each growth increment for male red mullets collected in the Southern Adriatic Sea. SD = standard deviation; CV = coefficient of variation.

N° Growth Increments	N° Specimens	Growth Increments						
		1°	2°	3°	4°	5°	6°	7°
1	284	4.6						
2	542	4.2	8.3					
3	544	4.4	8.7	11.2				
4	372	4.8	9.1	11.7	13.4			
5	120	4.9	9.2	11.8	13.8	15.2		
6	16	4.1	8.8	11.4	13.5	15.1	16.3	
7	1	4.9	8.7	10.9	13.1	14.9	16.7	17.8
Tot. number	1879	1879	1595	1053	509	137	17	1
Mean (cm)		**4.53**	**8.66**	**11.44**	**13.53**	**15.20**	**16.31**	**17.8**
Mean increment (cm)		5.34	3.31	2.81	2.11	1.71	1.14	
SD		0.73	0.94	1.13	1.14	1.22	1.13	
CV		1.36	1.02	0.92	0.80	0.82	0.70	

The first back-calculated TL was comparable to the length (4.4 cm) at which the morphological and ecological changes occurred in the juveniles, that is, in changing from pelagic ecophase to demersal ecophase. Consequently, the first supposed annuli can be considered as the false growth increment (demersal). Moreover, considering that the back calculated growth increments were laid down during the winter period, the ages assigned to these growth increments were as follows: 2° growth increment 0.5 years, 3° growth increment 1.5 years, 4° growth increment 2.5 years and so on. The growth parameters obtained by the back-calculation were as follows: L∞ = 28.824 cm, k = 0.171 year^−1^ and t_0_ = −1.747 year for females; L∞ = 22.077 cm, k = 0.321 year^−1^ and t_0_ = −1.173 year for males; L∞ = 33.543 cm, k = 0.154 year^−1^ and t_0_ = −1.634 year for combined sexes.

**Figure 10:**
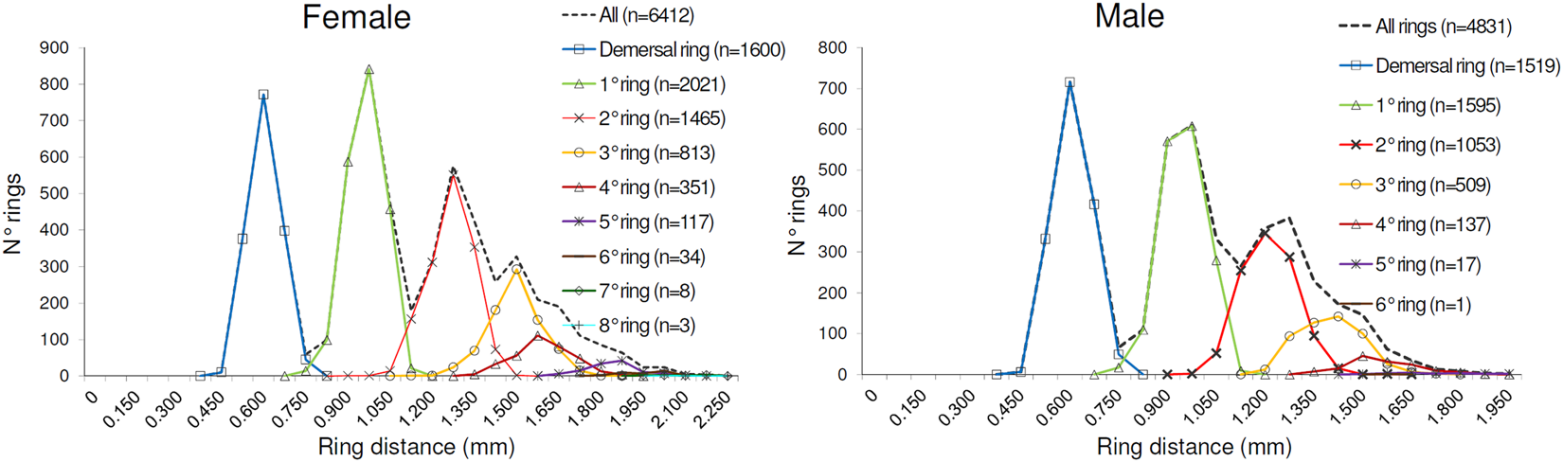
The frequency distributions by sex of the distance of the rings from the core (female specimens on the left and male specimens on the right).

The frequency distributions by sex of distances of the growth increments from the core (Fig. 3) are shown in Fig. 10.

**Figure 11:**
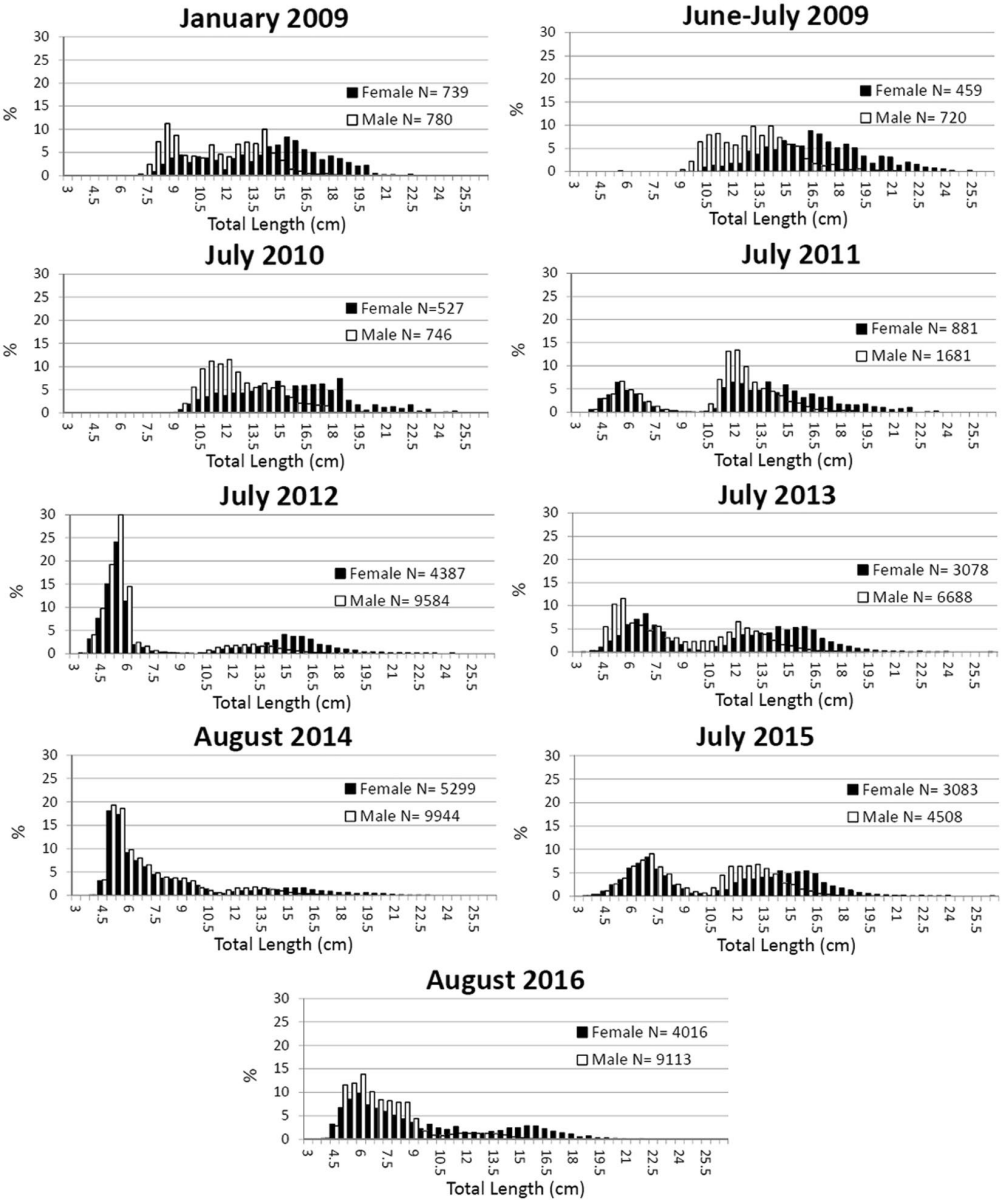
Length frequency distribution by sex of red mullet in the South Adriatic Sea.

The Bhattacharya method^31^ was used for the separation in the normal components of the length frequency distributions (Fig. 11) in the bottom trawl survey.

This method provided the mean length, standard deviation and the number of individuals in each modal component of the LFD. To each mode, a putative age was assigned according to the age scheme reported in Table 1. Hence, the lengths at age obtained were used to calculate VBGF growth parameter (GLM^23^): L_∞_ = 26.22 cm, k = 0.257 year^−1^ and t_0_ = −1.13 year for females; L_∞_ = 21.90 cm, k = 0.289 year^−1^ and t_0_ = −1.13 year for males; L_∞_ = 28.46 cm, k = 0.192 year^−1^ and t_0_ = −1.37 year for sexes combined.

The ELEFAN analysis of the LFD gave the following VBGF growth parameter: L_∞_ = 28.795 cm, k = 0.22 year^−1^ for females (Rn 0.203); L_∞_ = 22.48 cm, k = 0.39 year^−1^ for males (Rn 0.227); L_∞_ = 28.14 cm, k = 0.0.28 year^−1^ for combined sexes (Rn 0.257). The t_0_ calculated by the equation of Pauly were −0.895 years, −0.712 years and −0.805 years, respectively, for the female, male and combined sexes.

The statistical comparison between the mean back-calculated length of the annuli laid down in the winter and the mode identified in the LFD (GRUND 2009) from the period of transparent annuli deposition (Bhattacharya and ELEFAN) did not show significant differences (Kruskal-Wallis p > 0.05) among the age group identified (Table 4).

**Table 4:** Mean total lengths at age (cm) and standard deviation (SD) obtained by back-calculation formula, modal composition (Bhattacharya) and age slicing (ELEFAN) for females and males.

Age	FEMALE						MALE					
	Back-calculation		Bhattacharya		ELEFAN		Back-calculation		Bhattacharya		ELEFAN	
	Mean TL (cm)	SD	Mean TL (cm)	SD	Mean TL (cm)	SD	Mean TL (cm)	SD	Mean TL (cm)	SD	Mean TL (cm)	SD
0.5	9.32	1.01	9.36	0.73	9.4	0.88	8.66	0.94	8.75	0.48	8.79	0.57
1.5	12.73	1.17	12.95	0.58	12.62	1.65	11.44	1.13	11.55	0.52	11.29	1.01
2.5	15.49	1.20	15.64	0.97	15.42	1.04	13.53	1.14	13.59	0.73	13.93	0.87
3.5	17.49	1.25	17.75	0.61	18.26	1.06	15.2	1.22	15.1	0.63	15.01	1.03
4.5	19.29	1.18	19.66	0.6	19.59	1.28	16.34	1.13	16.25	0.7	16.26	1.09

**Figure 12:**
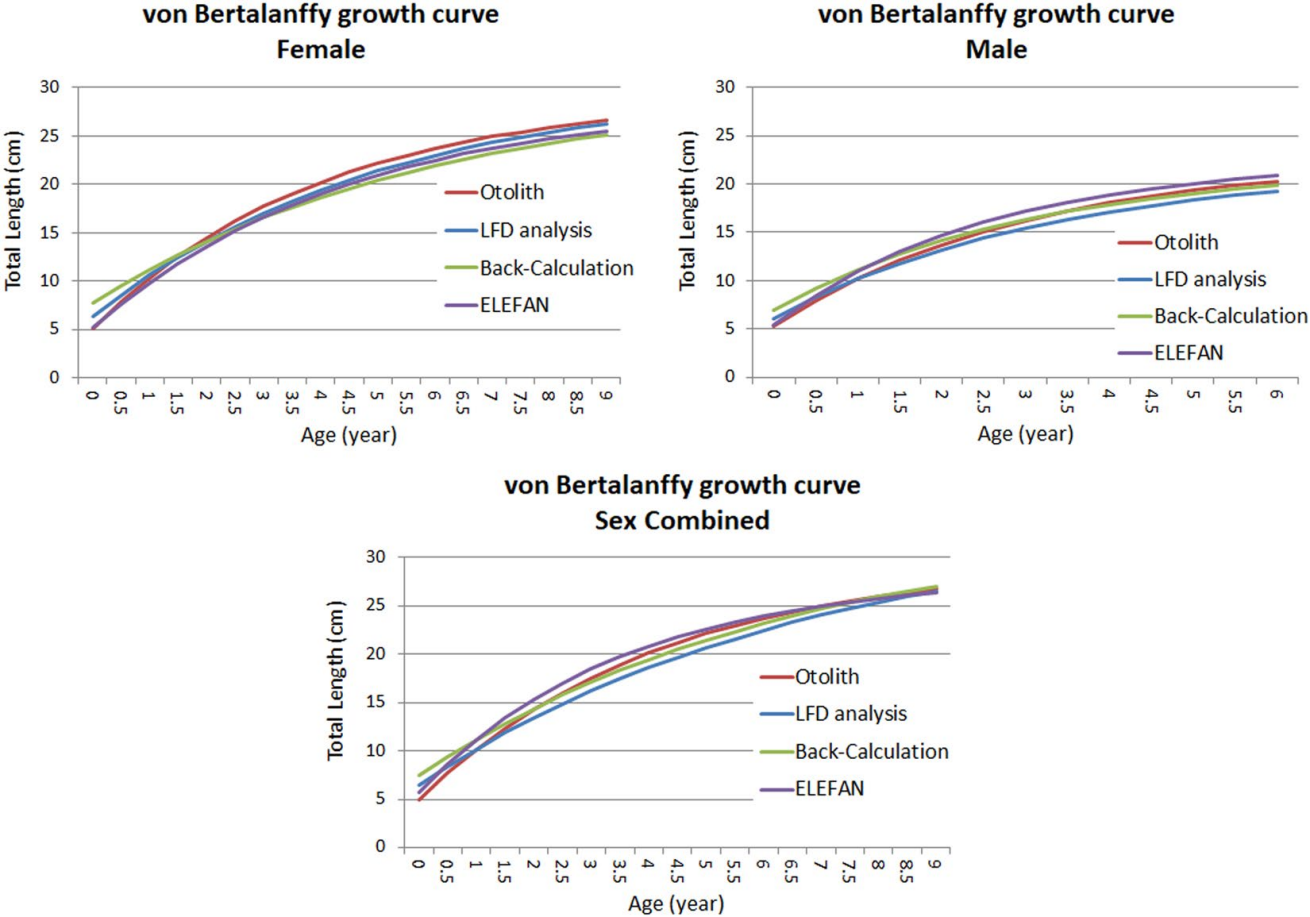
Growth curves obtained from otolith reading (red line), LFDA (blue line) and back-calculation (green line); ELEFAN (violet line) of female, male and combined sexes of *M. barbatus*.

The statistical comparison (Chen-test) of the VBGF growth curves from the otolith reading (back-calculation and direct age reading) and LFD analysis (Bhattacharya and ELEFAN methods) by sex did not show significant differences (Fig. 12) (F_obs_ > F_crit_).

**Figure 13:**
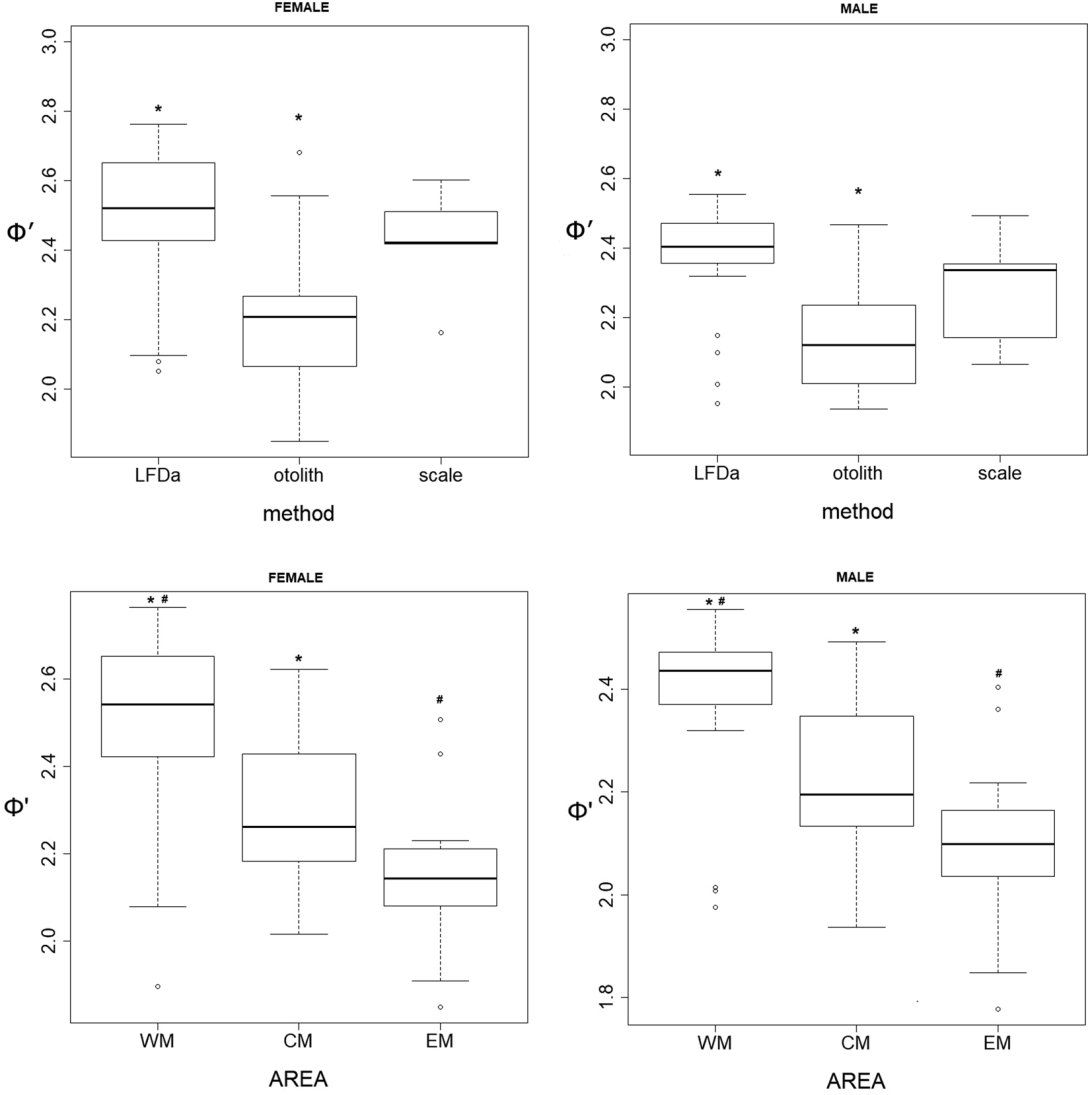
Box plot of Φ′ values for females (left) and males (right) grouped by method (LFD: length frequency distribution analysis; otolith reading; scale reading) and area (Mediterranean sub-region WM: Western Mediterranean; CM: Central Mediterranean; EM: Eastern Mediterranean). The symbols * and ^#^ indicate significant differences.

Figure 13 shows the box plot by sex of the Φ′ values (Table 5) grouped by method and Mediterranean sub-region. The Φ′ values from LFDA were significantly higher compared to those derived from the otolith reading for both sexes. The female Φ′ values calculated from the otolith reading ranged between 1.848^36^ and 2.686^37^, while those from LFDA ranged between 2.051^38^ and 2.763^8^. The male Φ′ values calculated from the otolith reading ranged between 1.937^39^ and 2.468^40^, while those from the LFDA ranged between 1.952^38^ and 2.554^41^. In addition, the Φ′ values grouped by area (Mediterranean sub-region WM: Western Mediterranean; CM: Central Mediterranean; EM: Eastern Mediterranean) showed significant differences. In particular, the Φ′ values from the WM were significantly higher than those from the CM and EM for both sexes.

**Table 5:** *M. barbatus* VBGF parameters, estimated length (cm) at age (from 0 to 8) and Φ′ by sex, Mediterranean sub-region and age estimation method.

References	Sex	Growth Parameters				Method	Area	Mediterranean Sub-region	Length (cm) at Age (year) calculated by growth parameters								
		L∞ (cm)	k (year^−1^)	t_0_ (year)	Φ′				0	1	2	3	4	5	6	7	8
Potoschi *et al*.^64^^##,^ *	F	22.89	0.15	−3.410	1.895	otolith	Northern coasts of Sicily	WM	9.17	11.08	12.72	14.14	15.36	16.41	17.31	18.09	18.76
Bianchini & Ragonese^57^	F	27.09	0.22	−1.570	2.208	otolith	Northern coasts of Sicily	WM	7.91	11.70	14.74	17.18	19.14	20.71	21.97	22.98	23.79
Sabatini *et al*.^40^	F	29.96	0.23	−1.540	2.315	otolith	Sardinia	WM	8.94	13.26	16.69	19.41	21.58	23.30	24.67	25.76	26.62
Sieli *et al*.^17^	F	22.12	0.38	−0.940	2.269	otolith	Gulf of Castellammare	WM	6.64	11.54	14.88	17.17	18.74	19.81	20.54	21.04	21.38
Canali^37^	F	25.5	0.74	−0.500	2.682	otolith	Tuscany coast	WM	7.89	17.10	21.49	23.59	24.59	25.06	25.29	25.40	25.45
Passalaigue,^65^	F	24.5	0.6	−0.200	2.556	otolith	Gulf of Lion	WM	2.77	12.57	17.96	20.91	22.53	23.42	23.91	24.17	24.32
Passalaigue,^65^	F	21	0.6	−0.400	2.423	scale	Gulf of Lion	WM	4.48	11.93	16.02	18.27	19.50	20.18	20.55	20.75	20.86
Spedicato *et al*.^52^	F	27.28	0.467	−0.410	2.541	LFDa	Central-Southern Tyrrhenian	WM	4.75	13.16	18.43	21.73	23.80	25.10	25.91	26.42	26.74
Voliani *et al*.^3 ###^	F	29.2	0.68	−0.537	2.763	LFDa	North Tyrrhenian	WM	8.93	18.93	24.00	26.56	27.86	28.52	28.86	29.03	29.11
Voliani *et al*.^3 ###^	F	27	0.7	−0.535	2.708	LFDa	North Tyrrhenian	WM	8.44	17.78	22.42	24.73	25.87	26.44	26.72	26.86	26.93
Voliani *et al*.^8^	F	28.1	0.69	−0.420	2.736	LFDa	North Tyrrhenian	WM	7.07	17.55	22.81	25.45	26.77	27.43	27.77	27.93	28.02
Voliani *et al*.^8^	F	26.5	0.64	−0.370	2.653	LFDa	North Tyrrhenian	WM	5.59	15.47	20.69	23.43	24.88	25.65	26.05	26.26	26.38
Voliani *et al*.^41^	F	27	0.697	−0.390	2.706	LFDa	Central Tyrrhenian	WM	6.43	16.75	21.90	24.46	25.73	26.37	26.69	26.84	26.92
SAMED^66^	F	27	0.396	−0.780	2.460	LFDa	Northern Adriatic	WM	7.17	13.66	18.02	20.96	22.93	24.26	25.16	25.76	26.17
SAMED^66^	F	29.1	0.53	−0.200	2.652	LFDa	Central-Southern Tyrrhenian	WM	2.93	13.69	20.03	23.76	25.96	27.25	28.01	28.46	28.72
SAMED^66^	F	24.5	0.6	−0.740	2.556	LFDa	Alboran Sea	WM	8.78	15.87	19.77	21.90	23.07	23.72	24.07	24.26	24.37
SAMED^66^	F	27.3	0.424	−0.424	2.500	LFDa	Catalan coast	WM	4.49	12.37	17.53	20.91	23.12	24.56	25.51	26.13	26.53
SAMED^66^	F	23.5	0.6	−0.200	2.520	LFDa	Corsica seas	WM	2.66	12.06	17.22	20.05	21.61	22.46	22.93	23.19	23.33
SAMED^66^	F	28.7	0.53	−0.200	2.640	LFDa	Sardinia seas	WM	2.89	13.51	19.76	23.44	25.60	26.88	27.63	28.07	28.33
SAMED^66^	F	29	0.358	−0.590	2.479	LFDa	Northern coasts of Sicily	WM	5.52	12.59	17.53	20.98	23.39	25.08	26.26	27.08	27.66
Greco *et al*.,^67^	F	26.7	0.168	−3.390	2.078	LFDa	Northern coasts of Sicily	WM	11.59	13.93	15.90	17.57	18.99	20.18	21.19	22.04	22.76
Present study	F	28.119	0.231	−1.189	2.262	otolith	South Adriatic	CM	6.75	11.16	14.66	17.43	19.64	21.39	22.78	23.88	24.75
Present study	F	29.824	0.171	−1.747	2.182	otolith b	South Adriatic	CM	7.70	11.18	14.11	16.58	18.66	20.42	21.89	23.14	24.19
Joksimović *et al*.^38^	F	29.131	0.122	−3.013	2.015	otolith	South-East Adriatic	CM	8.96	11.28	13.33	15.14	16.75	18.17	19.43	20.54	21.53
Tursi *et al*.^56^	F	24.5	0.28	−1.980	2.225	otolith	Western Ionian	CM	10.43	13.86	16.46	18.42	19.91	21.03	21.88	22.52	23.00
Andaloro & Prestipino Giarritta^39^	F	24.55	0.225	−2.010	2.132	otolith	Strait of Sicily	CM	8.93	12.08	14.59	16.60	18.20	19.48	20.50	21.32	21.97
Sonin *et al*.^49^	F	27.07	0.252	−0.950	2.266	otolith	Strait of Sicily	CM	5.76	10.51	14.20	17.07	19.29	21.03	22.37	23.42	24.23
Haidar,^68^	F	31.29	0.331	−0.950	2.511	scale-otolith	Croatia	CM	8.44	14.88	19.50	22.83	25.21	26.92	28.15	29.04	29.67
Gharbi & Ktary^69^##, *	F	30.61	0.279	−0.950	2.417	scale	Tunisian coast	CM	7.13	12.84	17.17	20.44	22.92	24.79	26.21	27.28	28.09
Gharbi & Ktary^69^^##,^ *	F	28.3	0.5	−0.040	2.603	scale	Tunisian coast	CM	0.56	11.48	18.10	22.11	24.55	26.02	26.92	27.46	27.79
Present study	F	26.469	0.257	−1.076	2.255	LFDa	South Adriatic	CM	6.39	10.94	14.46	17.18	19.29	20.92	22.17	23.15	23.90
Present study^###^	F	28.795	0.22	−0.895	2.261	LFDa	South Adriatic	CM	5.15	9.82	13.56	16.57	18.99	20.92	22.48	23.73	24.73
SAMED^66^	F	26	0.62	−0.200	2.622	LFDa	Strait of Sicily	CM	3.03	13.64	19.35	22.42	24.08	24.97	25.44	25.70	25.84
SAMED^66^	F	27.6	0.352	−0.848	2.428	LFDa	Ionian	CM	7.12	13.20	17.47	20.48	22.59	24.08	25.12	25.86	26.37
Joksimović *et al*.^38^	F	27.479	0.149	−2.688	2.051	LFDa	South-East Adriatic	CM	9.07	11.62	13.81	15.70	17.33	18.74	19.95	20.99	21.89
Sonin *et al*.^49^	F	20.51	0.276	−0.950	2.065	otolith	Israel coast	EM	4.73	8.54	11.42	13.62	15.28	16.54	17.50	18.22	18.78
Vassilopoulou & Papaconstantinou 1992^70^^#,^^+^	F	25.49	0.214	−2.134	2.143	otolith	Central Aegean	EM	9.34	12.45	14.97	16.99	18.63	19.95	21.02	21.88	22.58
Akyol *et al*.^71 #, +^	F	27	0.172	−1.844	2.098	otolith	Izimir Bay	EM	7.34	10.45	13.06	15.26	17.12	18.68	19.99	21.10	22.03
Livadas^72^	F	23.79	0.3	−0.950	2.230	otolith	Cyprus	EM	5.90	10.54	13.97	16.52	18.40	19.80	20.83	21.60	22.17
Papaconstantinou *et al*.^36^^+,^°	F	24.49	0.135	−2.941	1.908	otolith	Saranikos Gulf	EM	8.03	10.10	11.92	13.51	14.90	16.11	17.17	18.09	18.90
Papaconstantinou *et al*.^36^^+^ °	F	27.54	0.093	−4.302	1.848	otolith	Thermaikos Gulf	EM	9.08	10.72	12.21	13.58	14.82	15.95	16.97	17.91	18.77
Livadas^73^	F	28.4	0.18	−1.100	2.162	scale-otolith	Cyprus	EM	5.10	8.94	12.15	14.82	17.06	18.93	20.49	21.79	22.88
Hashem^74^	F	23.7	0.277	−0.950	2.192	ND	Egypt coast	EM	5.48	9.89	13.23	15.76	17.68	19.14	20.24	21.08	21.71
SAMED^66^	F	26.2	0.469	−0.310	2.508	LFDa	Crete	EM	3.55	12.03	17.33	20.65	22.73	24.03	24.84	25.35	25.67
SAMED^66^	F	30.3	0.292	−0.563	2.428	LFDa	Aegean sea	EM	4.59	11.10	15.96	19.59	22.31	24.33	25.84	26.97	27.81
Karlou-Riga & Vrantzas,^7 ###^	F	28.66	0.152	−1.138	2.096	LFDa	Saronikos Gulf	EM	4.55	7.95	10.87	13.38	15.53	17.39	18.98	20.34	21.51
Voliani *et al*.^41^	M	20.6	0.696	−0.600	2.470	LFDa	Central Tyrrhenian	WM	7.03	13.84	17.23	18.92	19.76	20.18	20.39	20.50	20.55
Spedicato *et al*.^52^	M	20.96	0.594	−0.250	2.417	LFDa	Central-Southern Tyrrhenian	WM	2.89	10.98	15.45	17.92	19.28	20.03	20.45	20.68	20.80
SAMED^66^	M	23.1	0.57	−0.200	2.483	LFDa	Central-Southern Tyrrhenian	WM	2.49	11.44	16.51	19.37	20.99	21.91	22.43	22.72	22.88
SAMED^66^	M	21	0.62	−0.740	2.437	LFDa	Alboran Sea	WM	7.73	13.86	17.16	18.93	19.89	20.40	20.68	20.83	20.91
SAMED^66^	M	22.1	0.506	−0.670	2.393	LFDa	Catalan coast	WM	6.35	12.61	16.38	18.65	20.02	20.85	21.34	21.64	21.83
SAMED^66^	M	23	0.394	−0.700	2.319	LFDa	Northern coasts of Sicily	WM	5.54	11.23	15.06	17.65	19.39	20.57	21.36	21.89	22.25
SAMED^66^	M	21.4	0.53	−0.500	2.385	LFDa	Corsica seas	WM	4.98	11.74	15.71	18.05	19.43	20.24	20.72	21.00	21.16
SAMED^66^	M	23.8	0.55	−0.200	2.494	LFDa	Sardinia seas	WM	2.48	11.50	16.70	19.71	21.44	22.44	23.01	23.35	23.54
Voliani *et al*.^3 ###^	M	22	0.74	−0.535	2.554	LFDa	North Tyrrhenian	WM	7.19	14.93	18.63	20.39	21.23	21.63	21.83	21.92	21.96
Voliani *et al*.^3 ###^	M	20.6	0.7	−0.553	2.473	LFDa	North Tyrrhenian	WM	6.61	13.65	17.15	18.89	19.75	20.18	20.39	20.50	20.55
Voliani *et al*.^8^	M	21.5	0.58	−0.780	2.428	LFDa	North Tyrrhenian	WM	7.82	13.84	17.21	19.10	20.16	20.75	21.08	21.26	21.37
Voliani *et al*.^8^	M	21.5	0.67	−0.440	2.491	LFDa	North Tyrrhenian	WM	5.49	13.31	17.31	19.35	20.40	20.94	21.21	21.35	21.42
Greco *et al*.,^67^	M	21.9	0.212	−2.100	2.007	LFDa	Northern coasts of Sicily	WM	7.87	10.55	12.72	14.47	15.89	17.04	17.97	18.72	19.33
Potoschi *et al*.^64^^##,^ *	M	21.77	0.62	−0.700	2.468	otolith	Northern coast of Sicily	WM	7.66	14.18	17.69	19.57	20.59	21.13	21.43	21.59	21.67
Passalaigue,^65^	M	19.07	0.26	−2.290	1.976	otolith	Gulf of Lion	WM	8.56	10.96	12.82	14.25	15.35	16.20	16.86	17.37	17.76
Canali^37^	M	22.5	0.56	−0.240	2.453	otolith	Tuscany coast	WM	2.83	11.26	16.08	18.83	20.41	21.30	21.82	22.11	22.28
Bianchini & Ragonese^57^	M	21.5	0.59	−0.800	2.436	otolith	Northern coasts of Sicily	WM	8.09	14.07	17.38	19.22	20.23	20.80	21.11	21.28	21.38
Sabatini *et al*.^40^	M	18.2	0.312	−2.330	2.014	otolith	Sardinia	WM	9.40	11.76	13.49	14.75	15.67	16.35	16.85	17.21	17.47
Passalaigue,^65^	M	16.8	0.8	−0.080	2.354	scale	Gulf of Lion	WM	1.04	9.72	13.62	15.37	16.16	16.51	16.67	16.74	16.77
Present study	M	21.848	0.285	−1.258	2.134	otolith	South Adriatic	CM	6.58	10.37	13.22	15.36	16.97	18.18	19.09	19.77	20.29
Present study	M	22.077	0.321	−1.173	2.194	otolith b	South Adriatic	CM	6.93	11.09	14.10	16.29	17.88	19.03	19.87	20.48	20.92
Tursi *et al*.^56^	M	22.4	0.27	−1.850	2.132	otolith	Western Ionian	CM	8.81	12.02	14.48	16.35	17.78	18.88	19.71	20.35	20.83
Sonin *et al*.^49^	M	22.52	0.339	−0.900	2.235	otolith	Strait of Sicily	CM	5.92	10.69	14.09	16.52	18.24	19.47	20.35	20.97	21.42
Andaloro & Prestipino Giarritta^39^	M	23.39	0.158	−2.840	1.937	otolith	Strait of Sicily	CM	8.46	10.64	12.50	14.09	15.45	16.61	17.60	18.45	19.17
Joksimović *et al*.^38^	M	17.811	0.282	−3.013	1.952	otolith	South-East Adriatic	CM	10.20	12.07	13.48	14.54	15.35	15.95	16.41	16.75	17.01
Present study	M	20.865	0.323	−1.014	2.148	LFDA	South Adriatic	CM	5.83	9.98	12.98	15.16	16.73	17.87	18.70	19.30	19.73
Present study^###^	M	22.48	0.39	−0.712	2.295	LFDA	South Adriatic	CM	5.45	10.95	14.67	17.19	18.90	20.06	20.84	21.37	21.73
Joksimović *et al*.^38^	M	17.811	0.282	−3.013	1.952	LFDa	South-East Adriatic	CM	10.20	12.07	13.48	14.54	15.35	15.95	16.41	16.75	17.01
SAMED^66^	M	20.3	0.602	−0.586	2.395	LFDa	Ionian	CM	6.03	12.49	16.02	17.96	19.02	19.60	19.91	20.09	20.18
SAMED^66^	M	20.2	0.64	−0.200	2.417	LFDa	Strait of Sicily	CM	2.43	10.83	15.26	17.59	18.83	19.48	19.82	20.00	20.09
SAMED^66^	M	23	0.43	−0.800	2.357	LFDa	Northern Adriatic	CM	6.69	12.39	16.10	18.51	20.08	21.10	21.76	22.20	22.48
Gharbi & Ktary^69^^##,^ *	M	25	0.497	−0.180	2.492	scale	Tunisian coast	CM	2.14	11.09	16.54	19.85	21.87	23.10	23.84	24.30	24.57
Gharbi & Ktary^69 ##,^ *	M	24.69	0.356	−0.900	2.336	scale	Tunisian coast	CM	6.77	12.14	15.90	18.53	20.38	21.67	22.57	23.21	23.65
Haidar,^68^	M	16.53	0.507	−0.900	2.142	scale-otolith	Croatia	CM	6.06	10.22	12.73	14.24	15.15	15.70	16.03	16.23	16.35
Vassilopoulou & Papaconstantinou^70^^#^^+^	M	22.71	0.25	−1.854	2.110	otolith	Central Aegean	EM	8.42	11.58	14.04	15.96	17.45	18.62	19.52	20.23	20.78
Sonin *et al*.^49^	M	15.59	0.473	−0.950	2.061	otolith	Israel coast	EM	5.64	9.39	11.73	13.18	14.09	14.66	15.01	15.23	15.36
Livadas^72^	M	19.67	0.426	−0.900	2.217	otolith	Cyprus	EM	6.26	10.91	13.95	15.94	17.23	18.08	18.63	18.99	19.23
Akyol *et al*.^71^^#, ^^+^	M	22.5	0.202	−2.299	2.010	otolith	Izimir Bay	EM	8.36	10.95	13.06	14.79	16.20	17.35	18.29	19.06	19.69
SAMED^66^	M	21	0.574	−0.330	2.403	LFDa	Crete	EM	3.62	11.21	15.49	17.89	19.25	20.01	20.45	20.69	20.82
SAMED^66^	M	23.8	0.405	−0.533	2.361	LFDa	Aegean sea	EM	4.62	11.01	15.27	18.11	20.00	21.27	22.11	22.67	23.05
Karlou-Riga & Vrantzas^7^^###^	M	21.5	0.271	−0.843	2.098	LFDa	Saronikos Gulf	EM	4.39	8.45	11.55	13.91	15.71	17.09	18.13	18.93	19.54
Livadas^73^	M	22	0.24	−1.200	2.065	scale-otolith	Cyprus	EM	5.51	9.02	11.79	13.97	15.68	17.03	18.09	18.93	19.58
Hashem^74^	M	19.52	0.333	−0.900	2.103	ND	Egypt coast	EM	5.05	9.15	12.09	14.19	15.70	16.78	17.56	18.11	18.51
Papaconstantinou *et al*.^36^^+^	M	19.23	0.191	−2.811	1.849	ND	Saranikos Gulf	EM	7.99	9.94	11.56	12.89	13.99	14.90	15.66	16.28	16.79
Papaconstantinou *et al*.^36^^+^	M	20.91	0.137	−4.251	1.777	ND	Thermaikos Gulf	EM	9.23	10.73	12.03	13.17	14.16	15.02	15.78	16.43	17.01
Canali^37^	U	24.5	0.74	−0.500	2.648	otolith	Tuscany coast	WM	7.58	16.43	20.65	22.66	23.62	24.08	24.30	24.40	24.45
Djabali *et al*.^3 ###^	U	29.7	0.21	−0.910	2.268	LFDa	Algerian coast	WM	5.17	9.81	13.58	16.63	19.11	21.12	22.74	24.06	25.13
Layachi *et al*.^75^	U	27	0.439	−0.090	2.505	LFDa	Nador	WM	1.05	10.27	16.21	20.05	22.52	24.11	25.14	25.80	26.23
Sanchez *et al*.^76^	U	29.7	0.09	−4.420	1.900	ND	Catalan coast	WM	9.75	11.46	13.03	14.47	15.78	16.98	18.07	19.07	19.99
Sanchez *et al*.^77^	U	33	0.38	−0.070	2.617	ND	Catalan coast	WM	0.87	11.02	17.97	22.72	25.97	28.19	29.71	30.75	31.46
Present study	U	29.008	0.194	−1.189	2.213	otolith	Adriatic Sea	CM	5.98	10.04	13.38	16.14	18.41	20.28	21.82	23.08	24.13
Present study	U	33.543	0.154	−1.634	2.239	otolith b	Adriatic Sea	CM	7.46	11.18	14.38	17.11	19.46	21.47	23.19	24.67	25.94
Ungaro *et al*.^78^	U	19.7	0.36	−1.180	2.145	otolith	South-West Adriatic	CM	6.82	10.71	13.43	15.33	16.65	17.57	18.21	18.66	18.98
Joksimović *et al*.^38^	U	30.1	0.118	−3.181	2.029	otolith	South-East Adriatic	CM	9.42	11.72	13.77	15.59	17.20	18.64	19.91	21.05	22.05
Jukić-Peladić & Vrgoč^79^	U	27.75	0.27	−0.616	2.318	LFDa	Crotia	CM	4.25	9.81	14.06	17.30	19.77	21.66	23.10	24.20	25.04
Joksimović *et al*.^38^	U	30.1	0.118	−3.182	2.029	LFDa	South-East Adriatic	CM	9.42	11.72	13.77	15.59	17.20	18.64	19.91	21.05	22.06
Present study	U	28.651	0.192	−1.318	2.198	LFDA	Adriatic Sea	CM	6.41	10.29	13.50	16.15	18.33	20.13	21.62	22.85	23.86
Present study^###^	U	28.14	0.28	−0.805	2.346	LFDA	Adriatic Sea	CM	5.68	11.16	15.31	18.44	20.81	22.60	23.95	24.98	25.75
Vrantzas *et al*.^15^	U	23.5	0.51	−0.860	2.450	otolith	Saranikos Gulf	EM	8.34	14.40	18.03	20.22	21.53	22.32	22.79	23.07	23.24
Togulga & Mater^80^^#,^^+^	U	26.471	0.1613	−2.702	2.053	otolith	Izimir Bay	EM	9.35	11.90	14.07	15.92	17.49	18.83	19.97	20.94	21.76
Akyol *et al*.^71^^#,^^+^	U	27	0.183	−1.506	2.125	otolith	Izimir Bay	EM	6.50	9.93	12.79	15.16	17.14	18.79	20.16	21.31	22.26
Kinacigil *et al*.^81^^#,^^+^	U	19.036	0.438	−0.777	2.201	otolith	Izimir Bay	EM	5.49	10.30	13.40	15.40	16.69	17.52	18.06	18.40	18.63
Çelik & Torcu^82^	U	26.08	0.127	−3.535	1.936	otolith	Edrmit bay	EM	9.43	11.42	13.17	14.71	16.06	17.26	18.31	19.24	20.05
Özbilgin *et al*.,^16^	U	24.26	0.565	−0.305	2.522	LFDa	Izimir Bay	EM	3.84	12.65	17.66	20.51	22.13	23.05	23.57	23.87	24.04
Gücü^83^	U	24.2	0.63	−0.569	2.567	LFDa	Iskenderun Bay	EM	7.29	15.19	19.40	21.64	22.84	23.48	23.81	23.99	24.09
Cicek,^84^	U	21.98	0.194	−1.168	1.972	LFDA	Iskenderun Bay	EM	4.46	7.55	10.09	12.19	13.91	15.34	16.51	17.47	18.27

Geographical area is also indicated. In the case of the data in Fork Length (^#^) or Standard Length (^##^) the length at age were transformed in in Total Length by the relationship reported in the paper (°) or by the following relationships: (*) Total Length = Standard Length/0.87 + 1.25^85^, (^+^) Total Length = Fork Length/0.96 + 1.78^85^. When t_0_ was not calculated by the authors, the empirical Pauly’s formula (^###^) was used^33^. F: female; M: male; U: unsexed; NA: not available; LFD a: length frequency distribution analysis; otolith b: back calculation; WM: Western Mediterranean; CM Central Mediterranean; EM: Eastern Mediterranean^63–65,67–85^.

## Discussion

Data on the deposition patterns for the transparent and opaque area on the otolith in the *M. barbatus* are very scarce^17^. In this study, the results of the MA highlighted the deposition of one opaque and one transparent zone per year. The opaque zone was laid down from June to November, and the transparent area was laid down from December until May. In addition, the MIA results confirmed that the transparent area was laid down from December to June, corresponding to the period of slow growth of otolith. These results are in agreement with the data (MA) reported for the southern Tyrrhenian Sea^17^.

One of the most important source of discrepancies between readers is the interpretation of the first winter annulus^9,10,12^ as it occurs in other species, such as hake^42^, horse mackerel^43^ and anchovy^44^. In particular, two different hypotheses have been proposed regarding the growth of red mullet: the slow-growing hypothesis (SGH) and the fast-growing hypothesis (FGH). In the first case (e.g. Lividas^32^; Sonin *et al*.^34^), only a false growth increment before the first annulus (winter area) was detected, reflecting the transition between the pelagic and the demersal phase (demersal ring). In the second case (e.g. Vrantzas *et al*.^15^; Sieli *et al*.^17^; Fiorentino *et al*.^35^), two false growth increments were identified before the first annulus: one was laid down during the pelagic phase (“pelagic ring”), and the second one was the “demersal ring”. In the Saronikos Gulf (Greece), Vrantzas *et al*.^15^ hypothesized the presence of transparent checks deposed in the summer (at 4–5 cm TL) and in the autumn (at 7.5–10 cm TL) in most of the young specimens. In the present study, the pattern of the deposition in the young specimens clearly showed that in the summer (July–August) there was a prevalence of otolith with transparent edges, but after September, the deposition pattern was comparable with that of the adult specimens. Based on these results, the second false growth increment did not occur. Moreover, the length of the passage from the pelagic to the demersal phase in the juveniles coincided with the back-calculated length of the first growth increment. Hence, in this study, only one false growth increment before the first annulus was considered as an age criterion in the otolith age estimation. These findings had an important effect on the age results^9^.

The juvenile red mullet is pelagic during the first weeks of life. Young red mullets live and feed near the surface until changes occur primarily in the mouth morphology, which includes the appearance of teeth and the development of the barbels^45^. The pelagic phase of *M. barbatus* is characterized by the blue livery of the specimens, which changes to a typical red livery when they move from the pelagic phase to the bottom of the sea^46^ during the settlement phase. The MEDITS net has a high vertical opening (3–4 m) in shallow water^18^, which allowed the capture of the both pelagic and demersal juveniles. These catches permitted the estimation of the settlement length and the clarification, for the first time, of some important life traits of juvenile *M. barbatus*.

During the pelagic phase *M. barbatus*, differ morphologically from specimens of *M. surmuletus* for the absence of a black or dark brown spot with an irregular shape on the first dorsal fin in the upper part as well as in varying proportions in the length of the snout^22^. Moreover, during the MEDITS survey (July–August), the settlement phase of the *M. surmuletus* was already completed^47^.

**Figure 14:**
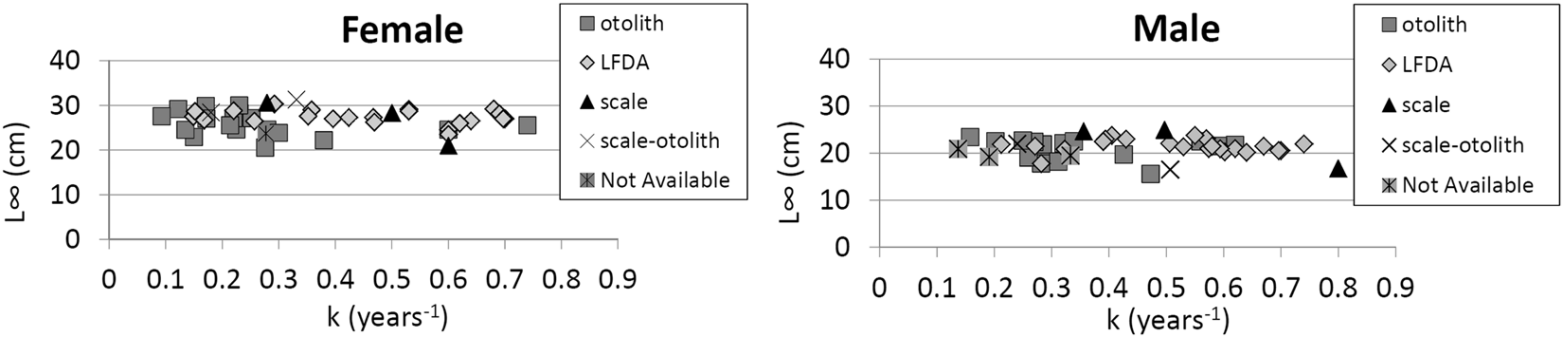
Plot of the Linf *vs* k (parameters of VBGF) from the literature classified by the methods applied.

The growth data (Table 5) in the Mediterranean basin showed a huge difference from the growth model. These differences could be caused by several factors: different sampling methodologies (commercial or survey)^48^, geographical differences^49^ (Fig. 13), age estimation criteria^9,11,17^, age estimation scheme^9^, material used (otolith or scale)^9^ (Fig. 13) and methodology (direct age estimation or LFDA)^9^ (Fig. 13). By plotting the Linf vs. the k (Fig. 14), it was possible to recognize the prevalence shown on the left of the graph (SGH with low k value) based on otolith reading. The LFDA is prevalent on the right of the graph (FGH with high k value).

According to the SGH, the Φ′ values (Table 5) ranged from 1.848 to 2.269 for the females and from 1.777 to 2.295 for the males. According to the FGH, the Φ′ values were between 2.315 to 2.763 for the females and 2.468 to 2.554 for the males. According to the SGH, the direct age estimation (otolith) was prevalent (Fig. 13). According to the FGH, the growth studies from LFDA was prevalent. Indeed, concerning the LFDA methods, the overlapping of the mode^39,50^ could represent an overestimation of the growth as the final results. However, the otolith reading could be biased by the false annual increments^21^. The outcomes of the present study (otolith reading and LFDA) seem in accordance with the SGH.

The Φ′ values grouped by method and geographical area were analysed and the results showed significant differences in both cases. Thus, the effects of the factors on determining the variability in the growth data in the Mediterranean basin could be combinatorial, making it difficult to determine the effect of a single factor.

The length-at-age data are fundamental in the application of analytical stock-assessment methods^21^. In addition, the uncertainties in the age data for red mullet are an obstacle to the proper management evaluation of this important resource^51,52^. Thus, the application of results of validation studies on age reading is crucial for the stock assessment of *M. barbatus*. Age validation should be a necessary step in all growth studies in order to improve accuracy and precision as well as to provide unbiased data for stock-assessment models.

The present work is the first to attempt a validation study of red mullet in the Mediterranean basin. In validation studies, two aspects shall be determined: (1) the increments are laid down according to a periodicity that can be related to a regular time scale (precision); (2) the age estimation structure has a consistent interpretable pattern (absolute age) of increments (accuracy)^13,21^. Both aspects have been poorly addressed in studies on *M. barbatus*^17^. Regarding the accuracy, Campana^13^ indicated the analysis of discrete length modes as a robust approach to validating the interpretation of annuli. The LFDA is based on the assumption that each age group has a normally distributed length. Hence, the modal lengths corresponding to age classes can be identified using different methods and then compared to individual lengths at age observed in the otolith reading^53^. The LFD (Fig. 11) in July and August showed an average mode of juveniles around 5 cm. The first mode in the winter months was an average of 9 cm. Similarly, it was possible to recognize the match between the other modes of LFD during the summer and winter months.

The comparison of the growth curves obtained from the otolith reading (back-calculation and direct age estimation) and the LFDA (ELEFAN and Bhattacharya methods) did not show any statistical differences. This result represented an indirect validation^13,21^ of the otolith age estimation criteria that were utilized.

A certain level of subjectivity^31^ is present in the Bhattacharya method compared with the ELEFAN. Nevertheless, the results of applying these methods were statistically comparable (Fig. 13). Indeed, the presence of the well-defined juvenile mode in some LFD surveys (Fig. 11) allowed the better interpretation of the mode discrimination and their following age assignment.

The back-calculation results were compared with the mean length of the mode (Bhattacharya method and ELEFAN) in the winter LFD (GRUND 2009). This analysis provided a further indirect validation of the detected age group, although it was limited to only one sampling occasion. The winter survey LFD was used in this analysis because the winter period seems to represent an age class (Figs 5 and 6).

In this study, the results of the growth pattern in the red mullets indicate that this species has a high growth rate in the first year, which is about 11 cm in the females and 10 cm in the males. Furthermore, the growth rate reduces in the following years to about 3–1.5 cm per year. This characteristic of red mullet growth seems to be in accordance with a quite biphasic growth pattern^50,54^. It is well known that growth depends on a complex interaction between energy allocation, foraging strategy, risk of predation, reproductive behaviour, short and long-term density dependence effects and the incidence of senescence. The characteristics of a high initial growth rate, precocious maturity^20^ and a reduction in growth thereafter^49,50^ could be explained by diversion of energy from somatic growth to reproduction, along with the general rule that minimum food intake occurs around and during the spawning period. The energy costs of reproduction represent an increasing strain on the metabolism of maturing and mature fish as they grow larger and older, decreasing the resources available for somatic growth^55^. This may also explain the difference in the growth between each sex in the red mullet (e.g. Tursi *et al*.^56^; Bianchini & Ragones^57^; Joksimović *et al*.^38^), indeed male of red mullets are in the reproductive deposition phase for almost the entire year^20^ with great effort spent of energy. The amount of energy allocated to growth and reproduction depends on a number of factors, some of them are intrinsic (genetic and physiological), others are environmentally driven (temperature and feeding). Thus, a compromise on energy balancing must exist reflecting the specific growth and reproduction dynamics in the lifetime of an individual fish^58^. Fisheries remove individuals at various trophic levels in the ecosystem affecting the distribution of energy and hence the amount of energy available for a particular fish. In this way fishery activities influence fish growth and maturation dynamics. This might also explain the contradictions which are evident among the high variability of red mullet growth (Fig. 6), despite their relatively high genetic homogeneity^59,60^.

In addition, the deposition pattern of the annuli reflected the growth pattern abovementioned. Indeed, the distance of the annulus represents about half of the otolith in the case of most of the old specimens in our sample (Fig. 10). Moreover, the decrease in distance between the annuli as the age estimation criteria to recognize the annuli is corroborated with the measurements of the distances from the nucleus (Fig. 10).

This study represents the first attempt of the age validation for the red mullet. The use of classical age validation methods (e.g. tag and recapture methods, chemical mark,, bomb radiocarbon dotation, captivity rearing)^13^ are hindered, considering the high mortality^14^ after the capture (stress, scale lost, wound) and the short life span of *M. barbatus*^16,51^. The results from different approaches, as well the MIA, MA, back-calculation, LFDA and morphological analysis, were analysed in a holistic perspective in order to validate the following age estimation criteria, used in the otolith reading:

- distance from the core of the consecutive annuli should be decreasing (Fig. 11);
- before the first annulus was laid down only one false growth increment (Fig. 5);
- deposition of one opaque and one transparent zone per year (Figs 5 and 6);
- transparent annuli should be visible more or less around the whole otolith in order to be considered as an *annulus*.

The sustainable exploitation for the stocks of red mullet is a key aim for fishery management. Stock assessment analysis can provide the precautionary reference points of fishing rates to prevent the overfishing and the collapse of the stock. Stock assessment techniques are highly dependent on availability and quality of the biological data, whether the aim is either long or short-term predictions. Information on growth parameters and/or Age Length Key are one of the most important input to obtain consistent outputs from the stock assessment models^61^. Indeed, age estimation errors, in some case, may also have contributed to errors in the populations assessment with the result of the collapse of the stocks^62,63^. Therefore, the results from this study offer a useful contribution to clarify the growth pattern of the red mullet and to overcome one of the impediments that may hamper the correct stock status diagnosis and the application of the appropriate management measures to prevent the collapse of the red mullet stock in the south Adriatic Sea.
